# Prognosis of patients with advanced bile tract carcinoma: assessment using the modified-Gustave Roussy Immune Score (mGRIm-s) as a clinico-immunological tool

**DOI:** 10.1007/s00432-024-05771-w

**Published:** 2024-05-09

**Authors:** Yue Ma, Yuting Pan, Yue Li, Huafang Guan, Guanghai Dai

**Affiliations:** 1 Medical School of Chinese PLA, Beijing, 100853 China; 2 Department of Medical Oncology, the First Medical Centre, Chinese PLA General Hospital, Beijing, 100853 China; 3 Department of Medical Oncology, the Fifth Medical Centre, Chinese PLA General Hospital, Beijing, 100039 China; 4 Yingtan City People’s Hospital, Yingtan, 335000 China

**Keywords:** Immunotherapy, Advanced bile tract carcinoma, The modified -Gustave Roussy Immune Score, Prognosis

## Abstract

**Background:**

The emergence of immune checkpoint inhibitors (ICIs) has enhanced survival outcomes for certain patients with advanced biliary tract carcinoma (BTC). Pinpointing those who would benefit most from immunotherapy remains elusive. We investigated the predictive value of the modified Gustave Roussy Immune Score (mGRIm-s) in BTC patients treated with ICIs.

**Methods:**

Data from 110 patients at Chinese People's Liberation Army General Hospital, spanning September 2015 to April 2021, were analyzed. The median follow-up duration was 38.7 months as of December 2023. Risk factors included low albumin, high lactate dehydrogenase, and an elevated neutrophil–lymphocyte ratio. Patients were stratified into low (patients with no risk factors) and high (patients with at least one risk factor) mGRIm-s groups based on these factors.

**Results:**

Survival outcomes post-immunotherapy favored the low mGRIm-s group, with significantly improved progression-free survival (PFS) and overall survival (OS) (8.50 months vs. 3.70 months and 21.60 months vs. 8.00 months). COX regression confirmed an elevated risk in the high mGRIm-s group. Subgroup analysis highlighted a notable survival advantage for low mGRIm-s patients receiving first-line immunotherapy.

**Conclusions:**

This study underscores mGRIm-s's potential in predicting immunotherapy response in BTC, paving the way for more targeted approaches.

**Supplementary Information:**

The online version contains supplementary material available at 10.1007/s00432-024-05771-w.

## Introduction

Biliary tract carcinoma (BTC) encompasses a range of aggressive tumors, including intrahepatic cholangiocarcinoma, periportal cholangiocarcinoma, distal cholangiocarcinoma, and gallbladder cancer. BTC account for less than 1% of all human cancers and have a low incidence in most developed countries (Vogel et al. 2023). However, cholangiocarcinoma is the second most commonly diagnosed primary liver cancer (10–15% of cases). The age-standardized incidence of cholangiocarcinoma in Europe, the United States, and Australia is 0.3–0.5 cases per 100,000 individuals. However, in regions where Schistosoma hepatica infection is endemic, such as Indochina, China, and Korea, the incidence of cholangiocarcinoma can be significantly higher, up to 40 times that of developed countries or regions (Banales et al. 2020). The mortality rate from bile duct cancer is also on the rise globally (Bertuccio et al. 2019).

Although radical surgery is the only curative treatment for BTC, unfortunately, most patients are found to have locally advanced or metastatic disease. Gemcitabine in combination with cisplatin is currently the only recommended first-line treatment for advanced or metastatic BTC. However, this chemotherapy regimen primarily offers palliative care and has a median survival of less than 1 year. Cancer treatment has entered the immune era with the growing research into tumor suppression by affecting the tumor immune microenvironment. In 2022, the TOPAZ-1 trial, a multicenter, double-blind, randomized phase III study, evaluated the combination of gemcitabine and cisplatin with the programmed death ligand 1 (PD-L1) inhibitor durvalumab in the therapy of BTC. The results demonstrated improved patient survival without significant additional toxicity compared to the control arm. Based on the results of the TOPAZ-1 trial, durvalumab in combination with gemcitabine plus cisplatin has been authorized as a first-line therapeutic approach for BTC (Kawamura et al. 2023; Lo et al. 2023; Rimini et al. 2023; Wheless et al. 2024). In addition, the programmed cell death protein-1 (PD-1) antibody pembrolizumab has been shown to be effective in the treatment of BTC as a second-line therapeutic treatment for cases of microsatellite instability (MSI). Both the single-arm, open-label Phase II clinical study (KEYNOTE-158) in advanced patients with 11 solid tumors and the global, multicenter, Phase Ib clinical study (KEYNOTE-028) in patients with PD-L1-positive advanced solid tumors reported anti-tumor efficacy of pembrolizumab. These studies suggest that pembrolizumab may be an effective treatment option for certain patients with advanced solid tumors, including BTC (Piha-Paul et al. 2020).

However, despite the fact that immune checkpoint inhibitors (ICIs) have revolutionized anticancer therapy, there is still a subset of patients who do not achieve lasting clinical benefit, and response rates to these immunologic agents are variable. This has significantly spurred the effort to actively search for reliable biomarkers that can accurately predict clinical outcomes. Studies have shown that neutrophil–lymphocyte ratio (NLR), prognostic nutritional index (PNI), platelet-lymphocyte ratio (PLR), albumin (ALB) levels and systemic immunoinflammatory index (SII) are linked to survival rates among tumor patients (Aslan et al. 2023; Cao et al. 2023; Huai et al. 2023; Mallardo et al. 2023; Muhammed et al. 2021; Pan et al. 2023; Zheng et al. 2023a).

Recent research on metrics for screening patients scheduled to receive immunotherapy has been centered on the Gustave Roussy Immune Score (GRIm-s), a comprehensive evaluation metric that incorporates lactate dehydrogenase (LDH), NLR and ALB. This score, which is predictive of immune efficacy, was first proposed in 2017 by Bigot F et al. and has subsequently been shown to be equally applicable in colorectal cancer, esophageal squamous cell carcinoma, resectable non-small cell lung cancer surgically, and gastric cancer in its advanced stage (Bigot et al. 2017; Li et al. 2020; Nakazawa et al. 2022; Shi et al. 2023; Tominaga et al. 2022). However, no pertinent studies have been documented in patients with BTC to date. Therefore, we investigated the clinical value of modified GRIm-s (mGRIm-s) in terms of efficacy and survival in BTC patients in this study.

## Methods

### Research subjects

Patients with advanced BTC who attended the Department of Medical Oncology of Chinese People's Liberation Army General Hospital from September 2015 to April 2021 were selected for this study. The median duration of follow-up was 38.7 months. Inclusion criteria: (1) Voluntary participation and written signed informed consent. (2) Individuals aged 18 years or older, with no restrictions on gender. (3) Pathologically confirmed diagnosis of BTC and have undergone comprehensive examinations including imaging, serum tumor marker analysis and histopathological examination. (4) Received at least two cycles of immunotherapy with ICIs. (5) Experienced at least one RECIST 1.1 evaluation of efficacy imaging during treatment. (6) Must receive routine blood and biochemical tests within seven days preceding the commencement of the first treatment with ICIs and have normal blood, coagulation, liver and kidney functions. Exclusion criteria: (1) Pregnant women or those who are lactating. (2) Patients who have been previously treated with ICIs. (3) Patients with any of the active autoimmune diseases or a past history of autoimmune disorders. (4) Patients who require treatment with corticosteroids (exceeding a dose equivalent to 10 mg/day of prednisone) or other immunosuppressive medications within 14 days prior to the initiation of study drug administration. (5) Those with other conditions that could affect peripheral blood ALB and neutrophils, such as hematological diseases, infections, viral hepatitis, cirrhosis etc. (6) Any condition that, in the judgment of the investigator, could potentially make treatment with the investigational drug hazardous, or that could hinder the assessment of the drug's efficacy, compromise the safety of the subject, or undermine the interpretation of the study results.

### Clinical information collection and evaluation

Clinical features of all patients included age, sex, Eastern Cooperative Oncology Group (ECOG) Performance Status (PS), smoking history, smoking exposure, drinking history, hypertensive disease, diabetes, location, pathological differentiation, pathological type, liver metastasis, distant metastasis, immunotherapy lines, immunotherapy regimen, neutrophil, LDH, ALB, and lymphocyte level in the 7 days before immunotherapy.

To evaluate treatment effectiveness, the Response Evaluation Criteria In Solid Tumors (RECIST) version 1.1 was utilized, encompassing four categories: complete response (CR), stable disease (SD), partial response (PR), and progressive disease (PD). Short-term efficacy was evaluated using the disease control rate (DCR), calculated as the sum of CR, PR and SD cases divided by the total number of cases multiplied by 100%. Additionally, the objective response rate (ORR) was determined by dividing the sum of CR and PR cases by the total number of cases, multiplied by 100%. Long-term efficacy was evaluated by assessing progression-free survival (PFS) and overall survival (OS).

### Treatment regimens

This study employed four treatment approaches: monotherapy with ICIs, combination therapy of ICIs with anti-angiogenic agents, combination therapy of ICIs with chemotherapy, and combination therapy of ICIs with both chemotherapy and anti-angiogenic agents.

The types and dosages of ICIs utilized in this study are outlined below: (1) Sintilimab was administered intravenously at a dose of 200 mg every 3 weeks; (2) Toripalimab was administered intravenously at a dose of 240 mg every 3 weeks; (3) Pembrolizumab was administered intravenously at the recommended dose of 3 mg per kilogram of body weight every 3 weeks; (4) Nivolumab was administered intravenously at the recommended dose of 2 mg per kilogram of body weight every 2 weeks. Nivolumab was initially evaluated after the third intravenous injection, which occurred 2–4 weeks after the start of treatment. In contrast, the evaluations of toripalimab, sintilimab, and pembrolizumab were conducted after the second intravenous injection, which occurred 3–5 weeks after the initial dosing.

Anti-angiogenic agents utilized in this study included lenvatinib (dosage range of 8–12 mg, administered orally once daily), apatinib (dosage of 850 mg, orally taken 30 min after meals, once daily), and bevacizumab (dosage of either 5 mg per kilogram of body weight every 2 weeks or 7.5 mg per kilogram of body weight every 3 weeks).

The chemotherapy regimens utilized in this study included the following: (1) the GS regimen, which consisted of tiggio (dosage of 40–60 mg, orally administered twice daily after breakfast and dinner for 14 consecutive days, followed by a 7-day break) combined with gemcitabine (dosage of 1250 mg per square meter of body surface area intravenously at the beginning of each cycle); (2) the GP regimen, involving cisplatin (dosage of 75 mg per square meter) and gemcitabine (dosage of 1250 mg per square meter intravenously at the beginning of each cycle); (3) the AS regimen, which combined conjugated albumin paclitaxel (dosage of 260 mg per square meter intravenously on the first day of each cycle) with tiggio (dosage of 40–60 mg, orally twice daily after breakfast and dinner for 14 consecutive days, followed by a 7-day break, with an intravenous injection on the first day of each cycle); (4) the GMOX regimen, consisting of oxaliplatin (dosage of 130 mg per square meter) combined with gemcitabine (dosage of 1250 mg per square meter intravenously on the first day of each cycle); (5) the AX regimen, which combined capecitabine (dosage of 1000 mg per square meter, orally twice daily after breakfast and dinner for 14 consecutive days, followed by a 7-day break) with conjugated albumin paclitaxel (dosage of 260 mg per square meter intravenously on the first day of each cycle); and (6) other regimens. The selection of these regimens was tailored to the pathological stage and overall health status of the patients. All patients provided informed consent for their treatment participation.

### Statistical analysis

Data processing and analysis were conducted utilizing R version 4.3.0 and SPSS version 26.0. Continuous variables were characterized using two metrics: median and standard deviation. Categorical variables were described through percentage indicators. Logistic regression models were employed to investigate the factors associated with mGRIm-s. Kaplan–Meier survival curves were constructed to analyze the significance of mGRIm-s in predicting OS and PFS. Additionally, COX regression was used to identify independent influencing factors for OS and PFS. Statistical significance was determined at the level of p < 0.05.

### Ethics statement

The present study protocol was reviewed and approved by the Ethics Committee Of Chinese PLA General Hospital (approval No.S2019-136–01). Informed consent was submitted by all subjects when they were enrolled.

## Results

### ROC analysis and grouping

The mGRIm-s was graded based on a combination of ALB, LDH, and NLR levels. In our study, we plotted receiver operating characteristic (ROC) curves to determine the optimal cut-off values for LDH at 184.2 U/L, ALB at 36.6 g/L, and NLR at 6.6. These cut-off values were determined using the pretreatment levels of these three indices as the test variable and death within 3 months of immunotherapy as the state variable. The results showed that the sensitivity of mGRIm-s, LDH, ALB, and NLR were 0.941, 0.588, 0.706 and 0.529, respectively, the specificity were 0.484, 0.677, 0.699 and 0.968, respectively, and the AUC were 0.713, 0.619, 0.719, and 0.755, respectively (Supplementary Fig. 1). Elevated LDH and NLR levels above their respective optimal cut-off values, as well as reduced ALB levels below the optimal cut-off, were identified as three distinct risk factors. The mGRIm-s was categorized into two groups based on the number of risk factors present. The group lacking any risk factors was designated as the low mGRIm-s group, while the group exhibiting one or more risk factors was designated as the high mGRIm-s group.

### Baseline characteristics and treatment status

**Fig. 1:**
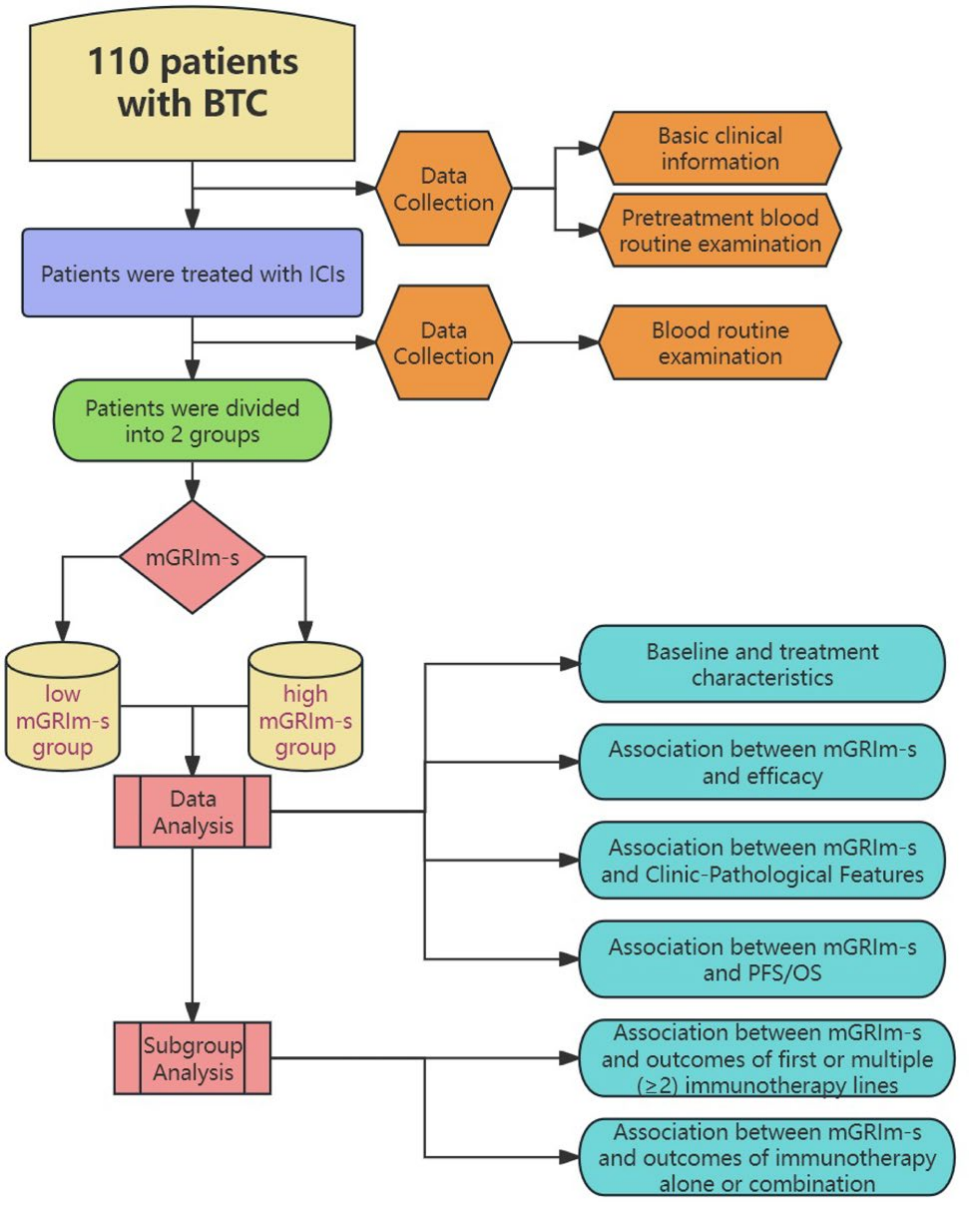
Study design flowchart. *BTC* bile tract carcinoma, *mGRIm-s* modified Gustave Roussy Immune Score, *ICIs* immune checkpoint inhibitors, *PFS* progression-free survival, *OS* overall survival

The study population selected for this study was 110 patients based on inclusion and exclusion criteria. The study's flowchart is presented in Fig. 1. All the baseline characteristics and treatment of patients with such conditions such as shown in Table 1. There were 46 BTC patients in the low mGRIm-s group, with a median age of 56 years, 26 males (56.52%), 20 females (43.48%), 46 patients (100.00%) ECOG PS 0–1 points. In terms of lifestyle habits, 14 (30.43%) of them had a history of smoking and 10 (21.74%) of them had more than 30 years of exposure to smoking and 15 (32.61%) had a history of alcohol consumption. In terms of past medical history, 36 (78.26%) had a history of surgery, 8 (17.39%) had a prior diagnosis of hypertension and 7 (15.22%) had a prior diagnosis of diabetes. Regarding tumor characteristics, 38 (82.61%) had tumors located in the bile ducts, 29 (45.31%) were moderate pathological differentiation, 43 (93.48%) were adenocarcinoma, 30 (65.22%) did not have liver metastases, and 33 (71.74%) had a number of distant metastases of 0–1. For immunotherapy, 7 (15.22%) had used chemotherapy prior to immunization and 6 (13.04%) had received chemotherapy combined with targeted therapy prior to immunization, 33 (71.74%) used first-line immunotherapy and 40 (86.96%) used immune combination therapy. There were 64 patients in the high mGRIm-s group, with a median age of 60 years, 41 males (64.06%), 23 females (35.94%), 6 patients (9.38%) with ECOG PS ≥ 2 points. Regarding lifestyle habits, 22 (34.38%) had a history of smoking and 13 (20.31%) had smoking exposure for more than 30 years and 18 (28.12%) had a history of alcohol consumption. In terms of past disease history, 40 (62.50%) had a history of surgery, 11 (17.19%) had a prior diagnosis of hypertension and 12 (18.75%) had a prior diagnosis of diabetes. In terms of tumor characteristics, 50 (78.12%) had tumors located in the bile ducts,  29 (45.31%) of the tumors became moderately pathologically differentiated, 62 (96.88%) were adenocarcinoma, 30 (46.88%) had liver metastases, and 26 (40.62%) had a number of distant metastases ≥ 2. In terms of immunotherapy, 13(20.31%) had undergone a chemotherapy-alone regimen preceding immunotherapy, while 5(7.81%) had been administered a chemotherapy regimen combined with targeted therapy before undergoing immunotherapy, 46 (71.88%) used first-line immunotherapy and 50 (78.12%) used immune combination therapy (Table 1).

**Table 1:** General data and clinical feature

Characteristics	Total (n = 110)	mGRIm-s		*p*
		Low (n = 46)	High (n = 64)	
Age, Mean ± SD	58.79 ± 9.91	56.72 ± 9.15	60.28 ± 10.23	0.062
Age				0.367
< 60	59 (53.64)	27 (58.70)	32 (50.00)	
≥ 60	51 (46.36)	19 (41.30)	32 (50.00)	
Sex				0.424
Female	43 (39.09)	20 (43.48)	23 (35.94)	
Male	67 (60.91)	26 (56.52)	41 (64.06)	
ECOG PS				0.087
0–1	104 (94.55)	46 (100.00)	58 (90.62)	
> 1	6 (5.45)	0 (0.00)	6 (9.38)	
Smoking history				0.664
No	74 (67.27)	32 (69.57)	42 (65.62)	
Yes	36 (32.73)	14 (30.43)	22 (34.38)	
Smoking exposure				0.856
0–30	87 (79.09)	36 (78.26)	51 (79.69)	
≥ 30	23 (20.91)	10 (21.74)	13 (20.31)	
Drinking history				0.613
No	77 (70)	31 (67.39)	46 (71.88)	
Yes	33 (30)	15 (32.61)	18 (28.12)	
Hypertensive disease				0.978
No	91 (82.73)	38 (82.61)	53 (82.81)	
Yes	19 (17.27)	8 (17.39)	11 (17.19)	
Diabetes				0.629
No	91 (82.73)	39 (84.78)	52 (81.25)	
Yes	19 (17.27)	7 (15.22)	12 (18.75)	
Surgical history				0.078
No	34 (30.91)	10 (21.74)	24 (37.50)	
Yes	76 (69.09)	36 (78.26)	40 (62.5)	
Location				0.562
Bile ducts	88 (80)	38 (82.61)	50 (78.12)	
Gall bladder	22 (20)	8 (17.39)	14 (21.88)	
Pathological differentiation				0.656
Low	50 (45.45)	19 (41.30)	31 (48.44)	
Medium	54 (49.09)	25 (54.35)	29 (45.31)	
High	6 (5.45)	2 (4.35)	4 (6.25)	
Pathological type				0.704
Non-adenocarcinoma	5 (4.55)	3 (6.52)	2 (3.12)	
Adenocarcinoma	105 (95.45)	43 (93.48)	62 (96.88)	
Liver metastasis				0.205
No	64 (58.18)	30 (65.22)	34 (53.12)	
Yes	46 (41.82)	16 (34.78)	30 (46.88)	
Distant metastasis				0.181
0–1	71 (64.55)	33 (71.74)	38 (59.38)	
≥ 2	39 (35.45)	13 (28.26)	26 (40.62)	
Pre-immunotherapy treatment				0.573
No treatment	79 (71.82)	33 (71.74)	46 (71.88)	
Chemotherapy alone	20 (18.18)	7 (15.22)	13 (20.31)	
Chemotherapy with targeted therapy	11 (10.0)	6 (13.04)	5 (7.81)	
Immunotherapy lines				0.988
First-line	79 (71.82)	33 (71.74)	46 (71.88)	
Multiline	31 (28.18)	13 (28.26)	18 (28.12)	
Immunotherapy regimen				0.236
Immunotherapy alone	20 (18.18)	6 (13.04)	14 (21.88)	
Immune-combination	90 (81.82)	40 (86.96)	50 (78.12)	

### The relationship between different mGRIm-s groupings and the short-term efficacy of immunotherapy.

**Fig. 2:**
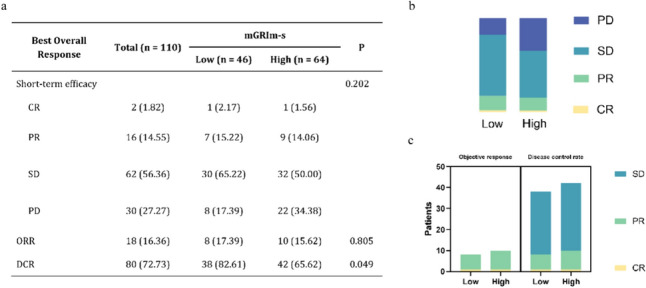
Relationship between low and high level groups of mGRIm-s and response to anti-PD-1 treatment. **a** Summary table of anti-PD-1 responses. **b** Proportion of anti-PD-1 responses. **c** Chart of anti-PD-1 response numbers. *PD-1* programmed cell death-1, *mGRIm-s* modified Gustave Roussy Immune Score, *CR* complete response, *PR* partial response, *SD* stable disease, *PD* progressive disease, *ORR* objective response, *DCR* disease control rate

The immunotherapy effects of all BTC patients were evaluated as follows: there were 2 CR, occupying 1.82%; 16 PR, occupying 14.55%; 62 SD, occupying 56.36%; and 30 PD, occupying 27.27%. In the evaluation of the composite index, the ORR was 16.36%, while the DCR was 72.73% (Fig. 2a). No statistically significant difference was observed in the proportional distribution of treatment efficacy between patients in the low mGRIm-s group and those in the high mGRIm-s group (Fig. 2b). Despite no significant difference in the proportional distribution of treatment efficacy, the proportion of patients achieving a DCR was significantly higher in the low mGRIm-s group compared to the high mGRIm-s group (82.61% vs 65.62%, *p* = 0.049) (Fig. 2a, c). After logistic regression model analysis, we inferred that immunotherapy lines and regimens were statistically associated with DCR (Table 2).

**Table 2:** Logistic regression of disease control rate

Patient Characteristics	Univariate Analysis OR (95% CI)	*p*	Multivariate Analysis OR (95% CI)	*p*
Age				
< 60	1.00 (Reference)			
≥ 60	1.22 (0.53–2.83)	0.640		
Sex				
Female	1.00 (Reference)			
Male	0.95 (0.40–2.24)	0.905		
ECOG PS				
0–1	1.00 (Reference)			
> 1	2.85 (0.54–14.99)	0.216		
Location				
Bile ducts	1.00 (Reference)			
Gall bladder	0.53 (0.16–1.72)	0.290		
Pathological differentiation				
Low	1.00 (Reference)			
Medium	0.28 (0.05–1.60)	0.153		
High	0.42 (0.08–2.31)	0.320		
Pathological type				
Non-adenocarcinoma	1.00 (Reference)			
Adenocarcinoma	646190241.6 (0.00–Inf)	0.999		
Liver metastasis				
No	1.00 (Reference)			
Yes	1.31 (0.56–3.06)	0.528		
Distant metastasis				
0–1	1.00 (Reference)			
≥ 2	1.59 (0.67–3.75)	0.292		
Immunotherapy lines				
First-line	1.00 (Reference)		1.00 (Reference)	
Multiline	4.00 (1.62–9.85)	0.003	2.89 (1.09–7.69)	0.033
Immunotherapy regimen				
Immunotherapy alone	1.00 (Reference)		1.00 (Reference)	
Immune-combination	0.13 (0.04–0.36)	< .001	0.16 (0.05–0.48)	0.001
mGRIm-s				
Low	1.00 (Reference)			
High	2.49 (0.99–6.25)	0.052		

### Association between mGRIm-s and clinical-pathological features

**Fig. 3:**
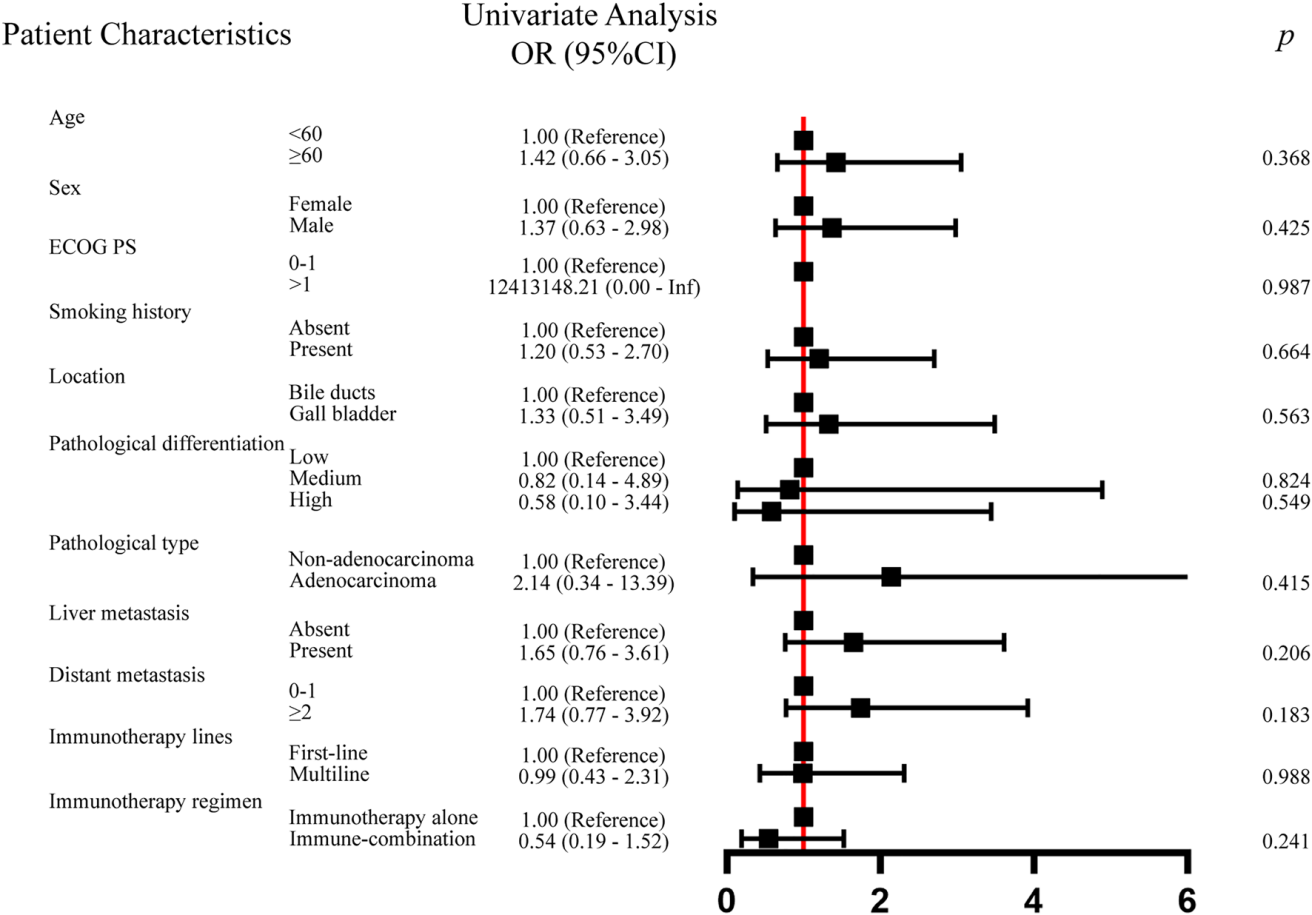
Clinical–pathological features of the patients according to the mGRIm-s. *mGRIm-s* modified Gustave Roussy Immune Score, *ECOG PS* Eastern Cooperative Oncology Group performance status score

We analyzed the statistical correlations between clinical-pathological characteristics and mGRIm-s using logistic univariate regression models, including Age, Sex, ECOG PS, Smoking history, Location, Pathological differentiation, Pathological type, Liver metastasis, Distant metastasis, Immunotherapy lines, Immunotherapy regimen. The results were shown in Fig. 3, and there was no statistically significant relationship between clinical-pathological characteristics and mGRIm-s.

### The relationship between mGRIm-s and long-term efficacy evaluation of tumors

**Fig.4:**
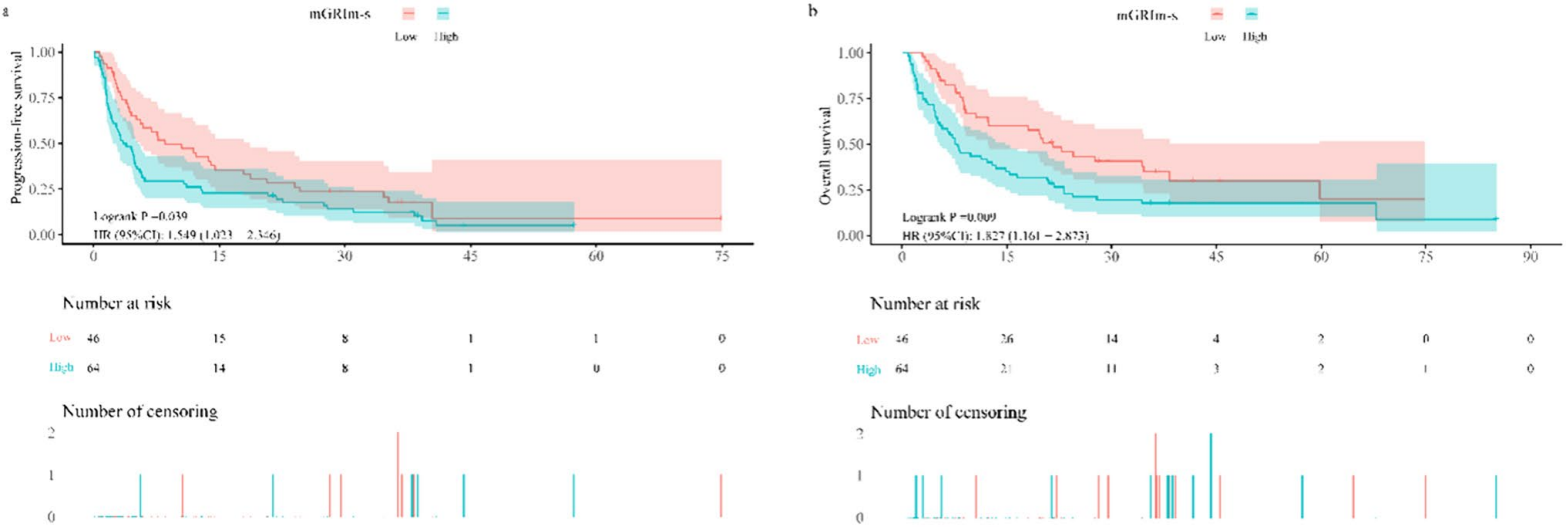
PFS (**a**, **b**) OS of BTC patients treated with PD-1 inhibitors. *PFS* progression free survival, *OS* overall survival, *BTC* bile tract carcinoma

**Fig. 5:**
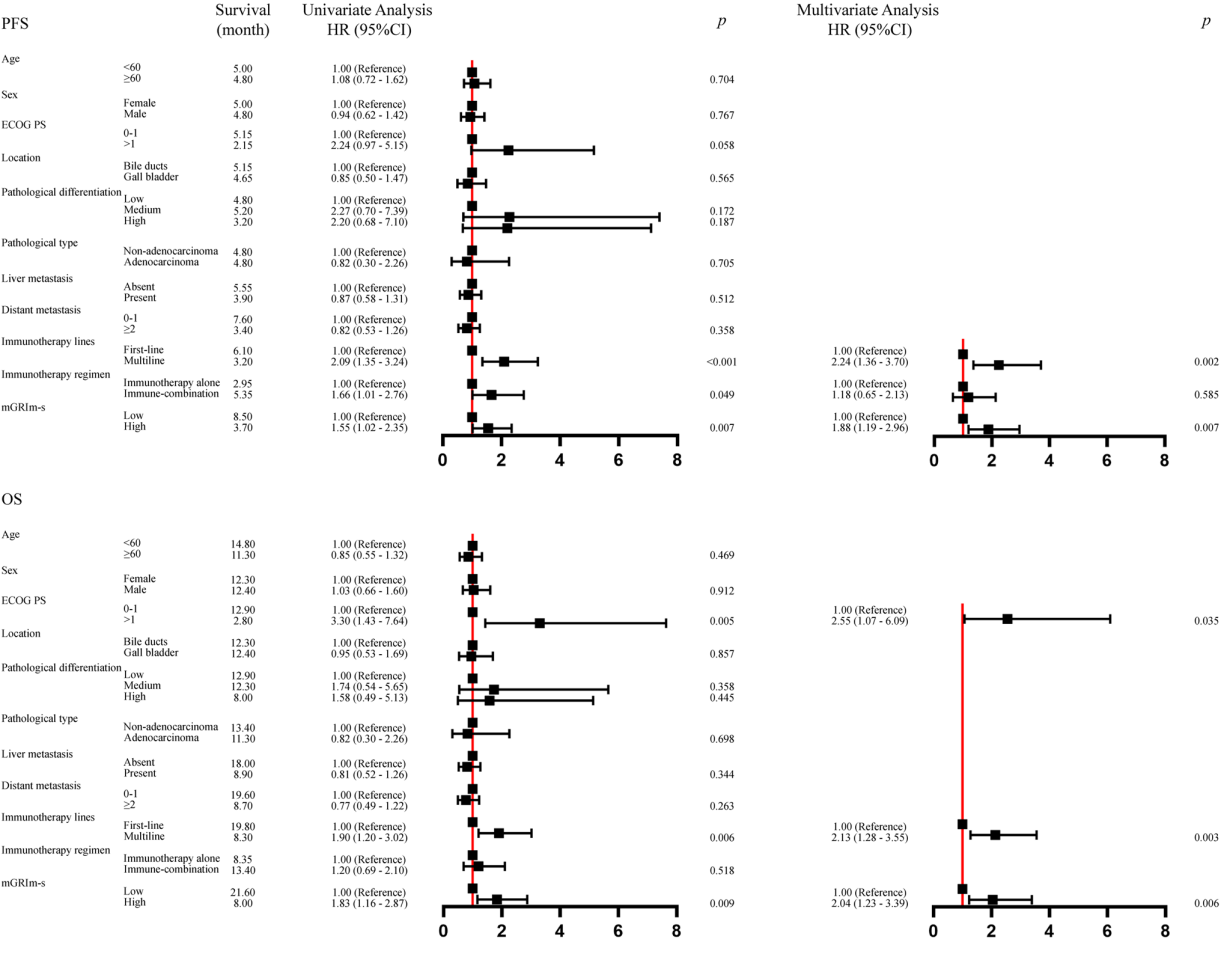
Univariate and multivariate analyses of factors associated with PFS and OS. *PFS* progression free survival, *OS* overall survival, *ECOG PS* Eastern Cooperative Oncology Group performance status scores, *mGRIm-s* modified Gustave Roussy Immune Score

The long-term treatment efficacy was assessed in 110 patients with BTC. By the final follow-up date of December 20, 2023, 94 (85.5%) had progressed and 81 (73.6%) had died. The analysis of patients' survival status and associated influencing factors is presented in Figs. 4 and 5.

Patients belonging to the low mGRIm-s group exhibited significantly enhanced PFS compared to those in the high mGRIm-s group (8.50 months vs. 3.70 months, *p* = 0.038) (Fig. 4a). The clinical-pathological related influences were analyzed by COX univariate regression modeling separately, and the three statistically significant indicators associated with PFS were screened out as Immunotherapy line, Immunotherapy regimen and mGRIm-s. A COX multivariate regression analysis was conducted to assess the three risk factors. The results indicated that patients in the high mGRIm-s group faced a 1.88-fold increased risk of disease progression compared to those in the low mGRIm-s group (HR = 1.88; 95% CI 1.19–2.96; *p* = 0.007) (Fig. 5). Furthermore, patients in the group receiving multiline immunotherapy exhibited a 2.24-fold elevated risk of disease progression compared to those treated with first-line immunotherapy (HR = 2.24; 95% CI 1.36–3.70; *p* = 0.002).

Patients belonging to the low mGRIm-s group exhibited a longer OS compared to those in the high mGRIm-s group (21.60 months vs. 8.00 months, *p* = 0.008) (Fig. 4b). The clinical-pathological related influences were analyzed by COX univariate regression modeling separately, and the three statistically significant metrics associated with OS were screened as ECOG PS, Immunotherapy lines and mGRIm-s. A COX multivariate regression analysis was conducted incorporating the three risk factors, revealing that the risk of mortality for patients in the high mGRIm-s group was 2.04 times higher compared to those in the low mGRIm-s group (HR = 2.04; 95% CI 1.23–3.39; *p* = 0.006) (Fig. 5). Furthermore, the risk of mortality among patients with an ECOG PS greater than 1 was 2.55 times higher compared to those with an ECOG PS of 0–1 (HR = 2.55; 95% CI 1.07–6.09; *p* = 0.035). The risk of mortality for patients receiving multiline immunotherapy was 2.13 times higher compared to those treated with first-line immunotherapy (HR = 2.13; 95% CI 1.28–3.55; *p* = 0.003).

### Subgroup analysis of mGRIm-s and lines of immunotherapy

According to the results of the analysis of the multivariate regression model presented in Fig. 5, the correlation between immunotherapy line and both PFS and OS was statistically significant. We performed a subgroup analysis of this to explore differences in survival among patients with the same immunotherapy line. Of the 79 individuals who received first-line immunotherapy, 33 (41.77%) belonged to the low mGRIm-s group and 46 (58.23%) to the high mGRIm-s group. As depicted in Supplementary Fig. 2, patients in the low mGRIm-s group exhibited significantly improved PFS of 14.00 months compared to 4.80 months in the high mGRIm-s group (*p* = 0.060), although the difference was not statistically significant. However, the OS was significantly prolonged in the low mGRIm-s group, reaching 24.50 months versus 11.30 months in the high mGRIm-s group (*p* = 0.043), indicating a statistically significant survival difference. Of the 31 individuals who received multiline immunotherapy, 13 (41.94%) were in the low mGRIm-s group and 18 (58.06%) were in the high mGRIm-s group. As shown in Supplementary Fig. 3, PFS (5.10 months vs. 2.30 months, *p* = 0.129) and OS (8.90 months vs. 6.10 months, *p* = 0.094) were significantly improved in patients in the low mGRIm-s group, but the survival differences in both PFS and OS were not statistically different.

### Subgroup analysis of combined immunotherapy with mGRIm-s

Since the initial proposal of mGRIm-s by Bigot et al. was based on immunobiotherapy included in early-stage trials, it did not consider immunotherapy combined with other treatments. Therefore, we divided the study population into two groups: those receiving immunotherapy alone and those receiving immunotherapy combined with other treatments. We then explored the relationship between mGRIm-s and immune prognosis in each group. Among the 20 patients (18.18%) who received immunotherapy alone, 6 patients (30.0%) belonged to the low mGRIm-s group, and 14 patients (70.0%) belonged to the high mGRIm-s group. As shown in Supplementary Fig. 4, patients in the low mGRIm-s group had significantly longer PFS compared to those in the high mGRIm-s group (3.4 months vs. 1.6 months, *p* = 0.065). Additionally, OS was also significantly improved in the low mGRIm-s group (9.1 months vs. 4.6 months, *p* = 0.358). On the other hand, among the 90 patients (81.82%) who received immunotherapy combined with other treatments, 40 patients (44.4%) had low mGRIm-s, and 50 patients (55.6%) had high mGRIm-s. As shown in Supplementary Fig. 5, PFS was improved in the low mGRIm-s group compared to the high mGRIm-s group (11.9 months vs. 4.6 months, *p* = 0.089), and OS was significantly extended in the low mGRIm-s group (21.6 months vs. 8.3 months, *p* = 0.020).

## Discussion

This study is the first research paper to evaluate the prognostic survival of BTC patients undergoing immunotherapy based on the mGRIm-s. The findings revealed a strong association between the mGRIm-s, serving as a combined biomarker of inflammation and nutrition, and survival outcomes in patients with BTC. In this study, we first plotted ROC curves using NLR, ALB, and LDH as indicators, and death at 3 months after receiving immunotherapy as an outcome indicator. Subsequently, calculations were conducted to determine the optimal cut-off values for each indicator. Specifically, the optimal cut-off values for NLR, ALB, and LDH were determined to be 6.6, 36.6, and 184.2, respectively. Upon determining the optimal cut-off values, we promptly identified LDH and NLR values exceeding the threshold, as well as ALB levels below the cut-off, as three distinct risk factors. Patients categorized as having a low mGRIm-s were those without any of these risk factors, while those with a high mGRIm-s exhibited one or more of the identified risk factors. In contrast, the recent study conducted by Lucy et al., which investigated the prognostic significance of mGRIm-s in patients with advanced pancreatic cancer, defined high mGRIm-s as the presence of two or more risk factors (Ma et al. 2023) While both our study and the one conducted by Lucy et al. demonstrated an association between high mGRIm-s and poor prognosis among patients, Lucy et al.'s findings, which were analyzed using a corrective model with multivariate regression, revealed that only male gender exhibited a significant correlation with high mGRIm-s. Whereas, in the present study, after analyzing by a univariate logistic regression model, the results showed that there were no clinical-pathological factors associated with mGRIm-s. Although the ORs of ECOG PS > 1 and high pathologic differentiation degree were extremely large in the present study, it was considered as a result of the small number of people who met the corresponding conditions.

Survival outcome analyses of patients in this study yielded shorter PFS (8.50 months vs. 3.70 months, *p* = 0.038) and OS (21.60 months vs. 8.00 months, *p* = 0.008) in patients with high mGRIm-s compared to patients with low mGRIm-s. The survival analysis conducted by Lucy et al. arrived at a similar conclusion, indicating that patients with high mGRIm-s had significantly shorter median OS compared to those with low mGRIm-s. (10.00 months vs. 6.40 months, *p* < 0.001). Furthermore, the present study sequentially conducted survival analyses employing both univariate and multivariate COX regression models. The findings from the multivariate COX correction model revealed that patients with high mGRIm-s exhibited a two-fold increased risk of mortality (HR = 2.04; 95% CI 1.23–3.39; *p* = 0.006) and an 1.8-fold higher risk of disease progression (HR = 1.88; 95% CI 1.19–2.96; *p* = 0.007) compared to those with low mGRIm-s. Patients who received immunotherapy as a multiline treatment exhibited a 2.1-fold increased risk of mortality (HR = 2.13; 95% CI 1.28–3.55; *p* = 0.003) and a 2.2-fold higher risk of disease progression (HR = 2.24; 95% CI 1.36–3.70; *p* = 0.002) compared to patients who underwent immunotherapy as first-line treatment. Subsequently, we conducted a subgroup analysis specific to the immunotherapy line, revealing a statistically significant reduction in OS among patients with high mGRIm-s who received immunotherapy as first-line treatment alone (24.50 months vs. 11.30 months, *p* = 0.043). This result also somewhat suggests that high mGRIm-s is associated with poor prognosis. In addition, our analysis also yielded a better prognosis for patients with low mGRIm-s than for those with high mGRIm-s regardless of whether immunotherapy was combined with chemotherapy, a result that is consistent with the previous conclusion that patients with low mGRIm-s have a poor prognosis. However, the correlation between mGRIm-s and OS was statistically significant only in patients who received immunotherapy in combination with chemotherapy, which should be considered as a reason for the insufficient number of cases for subgroup analysis. This study was also analyzed for short-term efficacy evaluation and showed that patients with low mGRIm-s had a DCR of up to 82.61%, which was much higher than the 65.62% DCR of patients with high mGRIm-s (*p* = 0.049).

The combination of gemcitabine and cisplatin has been the preferred first-line therapy based on the results of the ABC-02 trial reported in 2010 (Valle, et al. 2010). More recently, immunotherapy, especially in combination with chemotherapy, has also shown promising results. Sun and colleagues reported that patients with BTC who received immunotherapy in combination with chemotherapy had improved OS and PFS with no increase in toxicity compared to patients with BTC who received immunotherapy or chemotherapy alone (Sun et al. 2019). The promising outcomes observed in the TOPAZ-1 trial have led to the recommendation of using durvalumab in combination with gemcitabine and cisplatin as the preferred first-line treatment for patients with advanced or metastatic BTC (Fung and Syed 2023; Kang et al. 2022; Oh et al. 2023; Woods et al. 2022). Based on this study, the U.S. Food and Drug Administration (FDA), the European Medicines Agency (EMA), and the Chinese National Drug Administration (NMPA) approved durvalumab in combination with cisplatin + gemcitabine as first-line treatment for patients with previously untreated unresectable or metastatic BTC. The recent findings from the KEYNOTE-966 trial, which showed the effectiveness of pembrolizumab combined with gemcitabine and cisplatin in a similar patient population, further reinforce the promising potential of chemotherapy in combination with ICIs as a treatment option for advanced or metastatic BTC (Kelley et al. 2023; Zheng et al. 2023b). In our study, PFS and OS were similarly longer in patients with immune-combination chemotherapy than in patients with immunization alone, which is consistent with the findings of the above clinical trials.

In addition, an open-label, single-center, phase 2 study of the BTC clinical trial showed that patients in the chemotherapy plus durvalumab group and patients in the chemotherapy plus durvalumab and tremelimumab group had ORRs of 72% and 70%, respectively, which were higher than the ORRs of chemotherapy followed by chemotherapy plus durvalumab and tremelimumab group's (50%) (Oh et al. 2022). This gives the advantage of chemotherapy combined with immunotherapy in the first line. Our trial also subgrouped patients for first-line and multiple lines of immunotherapy, and the results continued to show a better prognosis with first-line immunotherapy. As research progressed, it became clear that the cisplatin plus gemcitabine plus durvalumab regimen also had variable effects in different genetic clusters. Rimini et al. divided patients treated with the above regimens into three genetic clusters, with the group of alterations in multiple pathways (of which DNA damage control, chromatin modification, RTK/RAS, cell cycle apoptosis, TP53 and PI3K were the most affected) had an ORR of up to 50%, which was superior to the group of mutations in the chromatin modification pathway as well as the group of alterations in the RTK/RAS and cell cycle apoptosis pathways (Rimini et al. 2024).

The response rate to BTC immunotherapy remains to be improved compared to other tumor types. This is partly due to the poor immune microenvironment and the relatively few diagnostic indications for biomarker-based immunotherapy for tumors (Fabris et al. 2021). Currently, the conventional method to assess whether cancer patients benefit from immunotherapy is to extract pathological sections and detect the expression of PD-L1, microsatellite instability (MSS), and tumor mutation burden (TMB) in their pathological tissues. Increasing numbers of researchers are committed to examining the substantial worth of simple, cost-effective, and actionable peripheral blood biomarkers in predicting the efficacy of immunotherapy. The mGRIm-s studied in this research is a composite index encompassing three peripheral blood biomarkers: NLR, ALB, and LDH. Individual studies have also reported on the predictive value of these biomarkers in determining the prognosis of immunotherapy among tumor patients.

Tumor-associated neutrophils (TANs) and lymphocytes are crucial constituents of the tumor immune microenvironment, playing a pivotal role in tumor-related immune responses (Fan et al. 2020; McFarlane et al. 2021). TANs play a significant role in facilitating tumorigenesis, metastasis, and drug resistance (Que et al. 2022). Recently, Ng Lai Guan et al. found that immature and mature neutrophils undergo reprogramming at the epigenomic, transcriptomic, and proteomic levels after entering the tumor and are transformed into T3 terminally differentiated neutrophils with high expression of dcTRAIL-R1, which then localize in the core region of the tumor tissues that are characterized by hyperglycolysis and hypoxia, and play a supportive effect on tumor growth in terms of promoting angiogenesis (Ng et al. 2024). Furthermore, PD-L1 + TANs suppress the production of interferon γ by CD8 + T cells, thereby preventing T cells from effectively eliminating tumor cells (Xue et al. 2022). Therefore, the higher the TAN, the worse the efficacy of immunotherapy. The primary constituents of lymphocytes, including T cells and natural killer cells, serve as the mainstay of the body's immune response. Lymphocytes are believed to positively influence the effectiveness of immunotherapy, implying that a higher lymphocyte count correlates with a stronger patient response to immunotherapy. On the other hand, the NLR is a complex biomarker of immune inflammation, and elevated NLR levels are strongly associated with a poor immune prognosis across various solid tumor types (Ouyang et al. 2023; Tang et al. 2021; Xiong et al. 2021).

Systemic inflammation triggers muscle atrophy associated with cachexia via activation of muscle nuclear factor-kappa B (NF-κB) signaling, leading to ubiquitin proteasome system (UPS)-mediated proteolysis (Argilés et al. 2005; Cai et al. 2004; Camps et al. 2006; Fearon et al. 2011). Low muscle mass is highly prevalent among patients with advanced cancer, which increases their susceptibility to toxic reactions during chemotherapy. Additionally, low muscle mass serves as an independent predictor of mobility and mortality in these patients (Prado et al. 2009; Prado et al. 2008; Tan et al. 2009). Of greater concern, cancer cachexia, which manifests as muscle wasting and weight loss, among others, is associated with poor immunotherapy efficacy, possibly due to the desensitizing effects of PD-1/PD-L1 inhibitors (Morimoto et al. 2021; Op den Kamp et al. 2012; Smith et al. 2011). The current study conducted by Melanie et al. indicates that ALB can serve as a crucial marker for evaluating cancer cachexia (Lipshitz et al. 2023) .In summary, we believe that ALB can be used as one of the indicators to comprehensively determine the prognosis of receiving immunotherapy by responding to the cancer cachexia of patients.

LDH is a key metabolic enzyme within the tumor microenvironment, facilitating the bidirectional conversion between pyruvate and lactate. It plays a crucial role in regulating energy metabolism in tumor cells. By altering the energy metabolic pathways of tumor cells, LDH acidifies the tumor microenvironment, leading to increased lactic acid extravasation into the tumor cell interstitium. This process promotes tumor cell proliferation, invasion, migration, drug resistance, and immune escape (de la Cruz-López et al. 2019). In addition, changes in energy metabolic pathways can help tumor cells adapt to the redistribution of energy substances in different environments, and also allow tumor cells to utilize the intermediates of the glycolytic process to achieve rapid proliferation (Brooks 2018; Liberti and Locasale 2016). LDH also regulates metastasis-associated proteins, reactive oxygen species (ROS), and activates the epithelial-mesenchymal transition (EMT) pathway. These actions are implicated in tumorigenesis, invasion, and metastasis (Miholjcic et al. 2023). Thus in oncology, elevated levels of circulating LDH have been considered a marker of poor prognosis.

There are inevitable limitations to this study: (1) Owing to the retrospective case analysis study design, which encompassed a restricted experimental sample size, the precision of the findings might be influenced by retrospective biases such as selection, recall, and measurement; (2) The data for the subjects was sourced exclusively from a single center; 3) The number of lines of immunotherapy and the specific regimen of the eligible patients varied; (4) The results of the indicators such as NLR, LDH, and ALB were affected by a variety of factors in clinical practice, and the cut-off values of the indicators were calculated based on previous reports; (5) This study was confined to the analysis of peripheral blood samples, and further assessment of clinical outcomes of patients with tumors should also be integrated with genomic and imaging tests; 6) Data from public domain databases or other institutions were not used for external validation to enhance the robustness of the data; (7) Due to the start of patient information collection in September 2015, when there were no standardized immunotherapy guidelines, most patients received chemotherapy prior to immunotherapy, and only a very small number of patients received the standard treatment regimen of durvacizumab in combination with cisplatin + gemcitabine; (8) The initial analysis did not strictly differentiate between patients treated with first-line immunotherapy and those treated with multiple lines of immunotherapy.

## Conclusions

This study demonstrated a statistically significant correlation between the composite biomarker mGRIm-s and OS, PFS, and DCR in patients with BTC receiving immunotherapy. Furthermore, the study suggested that patients with low mGRIm-s levels may derive benefit from immunotherapy. The utility of peripheral blood mGRIm-s as a prognostic biomarker that is both effective and cost-effective remains to be elucidated in larger-scale prospective studies. In future research, we aim to investigate effective immunotherapy markers from diverse perspectives, integrating genetic testing or immunohistochemistry results to achieve precise treatment for BTC. It is anticipated that this approach will lead to more BTC patients benefiting from the novel immunotherapy-based strategy.

## Supplementary Information

Below is the link to the electronic supplementary material.

Supplementary file1 (DOCX 515 KB)

## Data Availability

The data presented in this study are available upon request from the corresponding author. However, they are not publicly accessible at this time due to planned future analyses.

## Abbreviations

BTC: Biliary tract carcinoma
PD-L1: Programmed death ligand 1
PD-1: Programmed cell death protein-1
MSI: Microsatellite heightened instability
ICIs: Immune checkpoint inhibitors
NLR: Neutrophil–lymphocyte ratio
PLR: Platelet-lymphocyte ratio
SII: Systemic immunoinflammatory index
PNI: Prognostic nutritional index
ALB: Albumin
GRIm-s: Gustave Roussy Immune Score
LDH: Lactate dehydrogenase
mGRIm-s: Modified GRIm-s
ECOG: Eastern Cooperative Oncology Group
PS: Performance Status
RECIST: Response Evaluation Criteria In Solid Tumors
CR: Complete response
SD: Stable disease
PR: Partial response
PD: Progressive disease
DCR: Disease control rate
ORR: Objective response rate
PFS: Progression-free survival
OS: Overall survival
ROC: Receiver operating characteristic
MSS: Microsatellite instability
TMB: Tumor mutation burden
TANs: Tumor-associated neutrophils
NF-κB: Nuclear factor-kappa B
UPS: Ubiquitin proteasome system
ROS: Reactive oxygen species
EMT: Epithelial-mesenchymal transition
