# Supplementary material for: Prognosis of patients with advanced bile tract carcinoma: assessment using the modified-Gustave Roussy Immune Score (mGRIm-s) as a clinico-immunological tool
Source: J Cancer Res Clin Oncol. 2024 May 9;150(5):247. doi: 10.1007/s00432-024-05771-w (PMC11081983; doi:10.1007/s00432-024-05771-w)
Supplement: Supplementary file 1 — Supplementary file1 (DOCX 515 KB) [file 432_2024_5771_MOESM1_ESM.docx]

**Supplemental data**

**Prognosis of Patients with Advanced Bile Tract Carcinoma: Assessment Using the Modified-Gustave Roussy Immune Score (mGRIm-s) as a Clinico-Immunological Tool**

Yue Ma; Yuting Pan ; Yue Li; Huafang Guan; Guanghai Dai

**Contents**

Supplemental Figures 3

Figure S1. Summary of the results of selecting the optimal cut-off value through the ROC curve... 3

Figure S2. Survival analysis of BTC patients receiving first-line immunotherapy.... 3

Figure S3. Survival analysis of BTC patients receiving multiline immunotherapy... 4

Figure S4. Survival analysis of BTC patients receiving immunotherapy alone... 4

Figure S5. Survival analysis of BTC patients receiving immunotherapy combined with other treatments... 5

# Supplemental Figures

| **Figure S1:** Summary of the results of selecting the optimal cut-off value through the ROC curve. |
| --- |
| **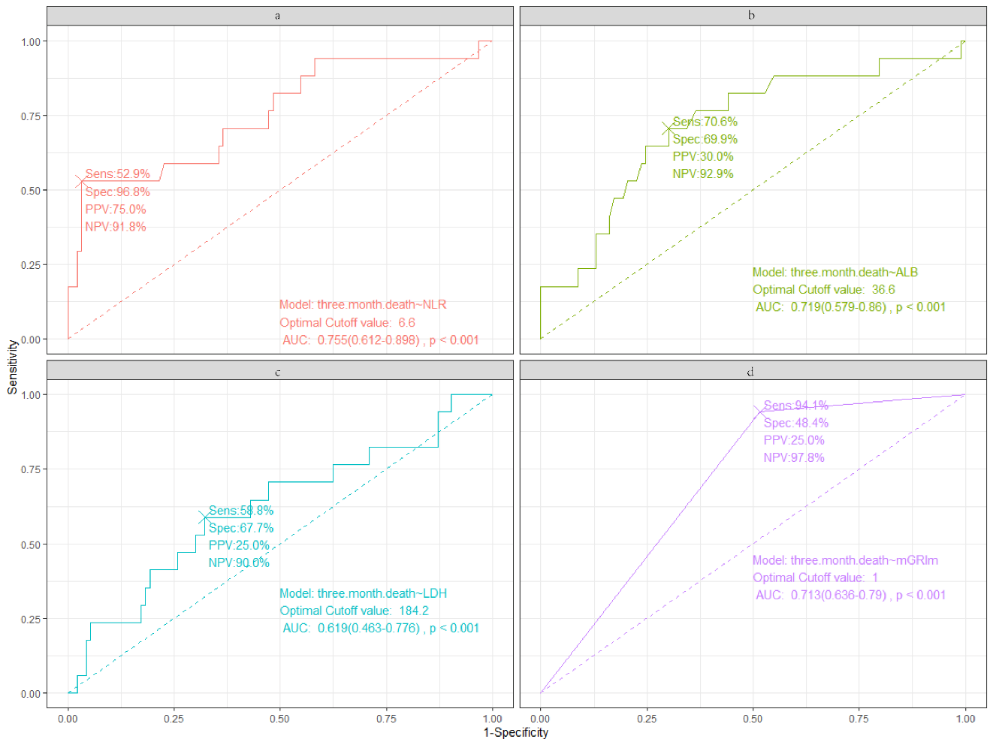** |
| ROC curves for pretreatment NLR (a), ALB (b), LDH (c) and mGRIm-s (d) to assess 3-month death rates. Abbreviations: ROC: receiver operator characteristic; mGRIm-s: modified Gustave Roussy Immune Score; Sens: sensitivity; Spec: specificity; PPV: positive predictive value; NPV: negative predictive value. |

| **Figure S2:** Survival analysis of BTC patients receiving first-line immunotherapy. |
| --- |
| **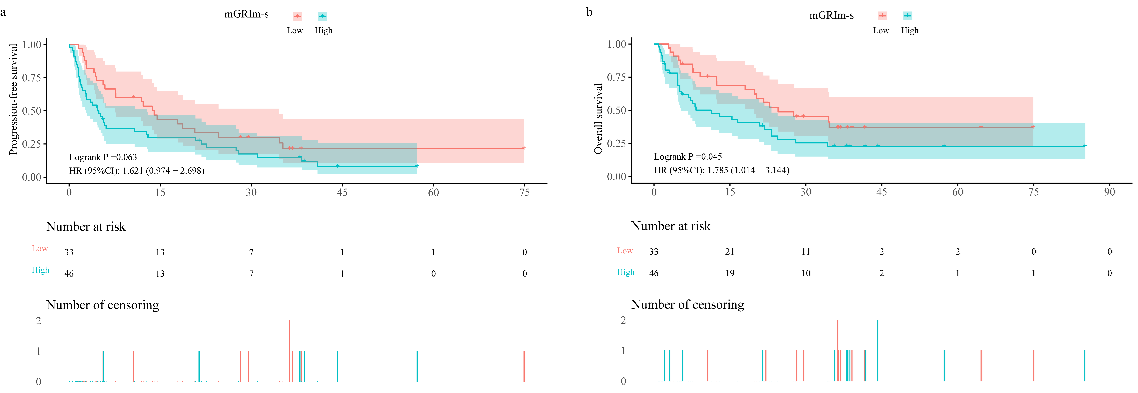** |
| PFS (a) and OS (b) in first-line BTC patients treated with PD-1 inhibitors. Abbreviations: PFS: progression free survival; OS: overall survival; BTC: bile tract carcinoma; PD-1: programmed cell death-1. |

| **Figure S3:** Survival analysis of BTC patients receiving multiline immunotherapy. |
| --- |
| **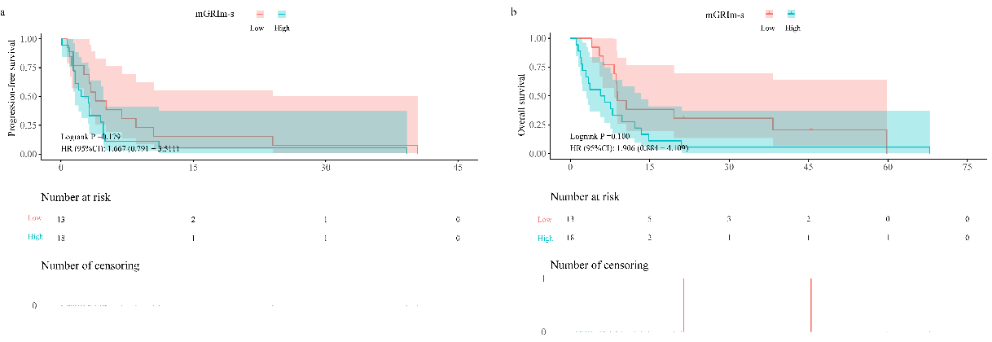** |
| PFS (a) and OS (b) in multiline BTC patients treated with PD-1 inhibitors. Abbreviations: PFS: progression free survival; OS: overall survival; BTC: bile tract carcinoma; PD-1: programmed cell death-1. |

| **Figure S4:** Survival analysis of BTC patients receiving immunotherapy alone. |
| --- |
| **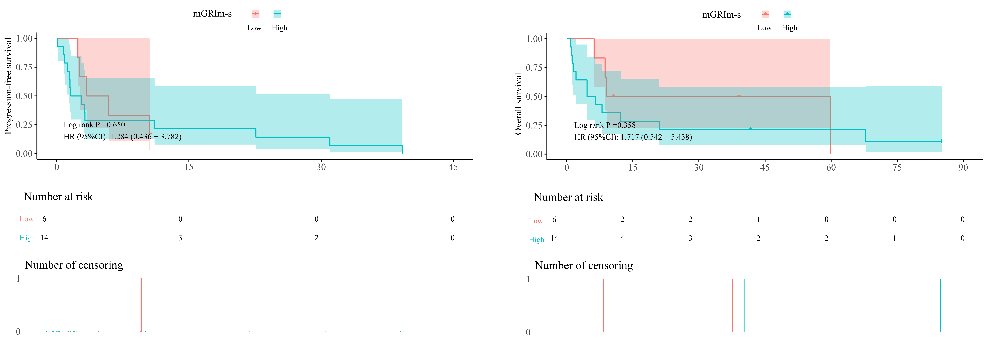** |
| PFS (a) and OS (b) in BTC patients treated with PD-1 inhibitors alone. Abbreviations: PFS: progression free survival; OS: overall survival; BTC: bile tract carcinoma; PD-1: programmed cell death-1. |

| **Figure S5:** Survival analysis of BTC patients receiving immunotherapy combined with other treatments. |
| --- |
| **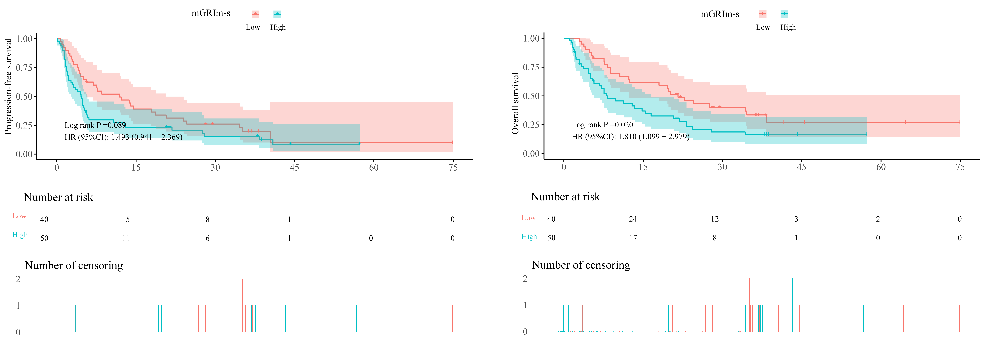** |
| PFS (a) and OS (b) in BTC patients treated with PD-1 inhibitors combined with other treatments. Abbreviations: PFS: progression free survival; OS: overall survival; BTC: bile tract carcinoma; PD-1: programmed cell death-1. |
